# 

**Published:** 1891-06

**Authors:** 


### CONTENTS:

Article I.—Twenty-third Annual Session of the Georgia State Dental Society. - - - - - - - 49

" II.—Observations on Bromide of Ethyl in Dental Surgery. By J. F. W. Silk, M. D. - - - - - - 60

" III.—Infantile Paralysis. By A. van Hoff Gosweiler, A. M., M. D. - - - - - - - - 65

" IV.—Compound Fracture of the Alveolus and Maxillary. By C. H. Wells, L. D. S. - - - - - - 70

### EDITORIAL.

William F. Wegge, M. D., D. D. S. - - - - - 74

Monthly Meeting of the American Academy of Dental Science. 74

The Death of Dr. Edward Maynard. - - - - - 75

### OBITUARY.

James William White, M. D., D. D. S., A. M. - - - - 75

Dr. Edward Maynard. - - - - - - - 86

William H. Atkinson, M. D., D. D. S. - - - - - 91

Dr. Henry R. Shine. - - - - - - - 93

### MONTHLY SUMMARY.

Amputation of the Forearm under Cocaine. - - - - 95

Sensitive Dentine. - - - - - - - 96

